# High Efficiency of the Graphene-Decorated Boron Nitride for Dye Removal from Aqueous Solution: Modeling and Optimization Process Designed for Textile Wastewater Treatment

**DOI:** 10.1021/acsomega.5c07579

**Published:** 2025-10-24

**Authors:** Romuald Teguia Doumbi, Paulino Vasco Mariano Muguirrima, Artur de Morais, Felipe Bohn, Carlos A. Martínez-Huitle, Igor Cretescu, Marcio Assolin Correa

**Affiliations:** † Postgraduate Program in Materials Science and Engineering, Federal University of Rio Grande do Norte, Natal, Rio Grande do Norte 59078-970, Brazil; ‡ Department of Physics, Federal University of Rio Grande do Norte, Natal, Rio Grande do Norte 59078-970, Brazil; § Renewable Energies and Environmental Sustainability Research Group, Institute of Chemistry, Federal University of Rio Grande do Norte, Natal, Rio Grande do Norte 59078-970, Brazil; ∥ “Cristofor Simionescu” Faculty of Chemical Engineering and Environmental Protection, “Gheorghe Asachi” Technical University of Iasi, 73 Blvd, Mangeron, Iasi 700050, Romania

## Abstract

This study reports the use of graphene-decorated boron nitride (GBN) as an efficient adsorbent for azo dyes, with the aim of treating textile wastewater. GBN was synthesized by a facile route and characterized by XRD, SEM/EDS, TGA-DTA, and BET analyses, revealing a microporous and crystalline structure. Methyl orange (MO) was adopted as a model dye, and batch adsorption experiments were optimized by using response surface methodology (RSM) with pH, adsorbent mass, and initial MO concentration as variables. Results showed that the pH and adsorbent mass were the main factors influencing MO removal. Under optimal conditions (pH 2, 0.062 g of adsorbent, 100 mg/L MO), GBN reached a high adsorption capacity of 322.5 mg/g in 20 min and retained good performance after 10 reuse cycles. The synergistic properties of graphene and boron nitride make GBN a promising material for efficient textile wastewater treatment.

## Graphical abstract

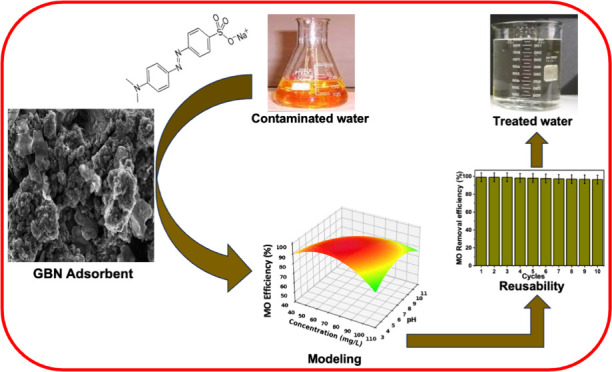

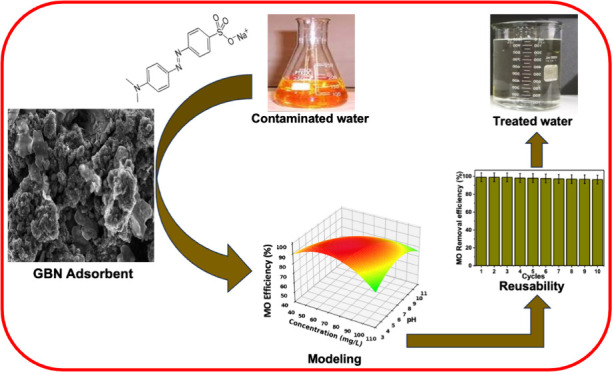

## 1 Introduction

The importance of water in maintaining life on the planet is undeniable. Most human activities require the use of water, which can lead to contamination of this important resource. Consequently, this contamination affects humans and other living beings, bringing with it several socioeconomic impacts. Therefore, the use of this resource must be considered to not harm any of its different uses for human life. For instance, the toxicity and load of industrial pollutants have more significant impacts than the volume of these waters and can even generate acid rain. For this reason, international laws establish limits for the release of various substances into bodies of water.^1^

For instance, the textile industry is one of the largest polluters in the world, negatively impacting the environment through the excessive use of natural resources (water, land, among others) and various chemical products.^2^ Pollution of water resources by industrial organic compounds, specifically synthetic dyes, has been commonly reported, causing several problems to human and animal health.^3^ Annually, half of the 800 million tons of synthetic dyes produced are of the “azo” type, which may present carcinogenic traits.^4^ In particular, methyl orange (MO) is a typical synthetic dye of an anionic nature with azo bonds (−N=N−) in the aromatic structure. It generally presents high stability to photochemical processes and degradation by natural factors, implying considerable difficulty in removing it from wastewater.^5^ Its extensive use can cause contamination of the aquatic environment, giving color to the process wastewater.^6–8^

Actually, the available water treatment techniques used to remove dyes from industrial wastewater include biological treatment, coagulation, sedimentation, adsorption, advanced oxidation processes (AOPs),^9,10^ and membrane separation.^11–15^ Among the distinct techniques, the adsorption appears to be the most attractive of all the methods due to its simple design, easy operation, low operating cost, and being free from the generation of toxic byproducts.^16,17^ Nowadays, several adsorbents are being used for dye removal from aqueous solutions, such as activated carbon, zeolites, silica gel, alumina, and graphene nanosheets.^18–22^

Within this context, graphene emerges as a great candidate to compose the base material for the absorption process. Graphene-based materials (GBMs), such as graphene oxide and pristine graphene, have great potential for the removal of pharmaceuticals,^6,16,23^ such as atenolol, ciprofloxacin, carbamazepine, ibuprofen, among others.^6^ Moreover, nanostructured porous graphene (NPG) is an ideal water treatment material because of its excellent hydrophobicity, adsorption capacity, recyclability, and low toxicity.^24^

In particular, the high specific surface area can result in a lower filter volume for water treatment, and its high process efficiency will lead to a lower regeneration frequency.^16,24,25^ However, modification of graphene with functional groups can further enhance its selectivity and adsorption capacity.^26^ Thus, in order to use low-cost materials, some works report the combination of graphene with other materials, such as magnetic, metals, and ceramic-based materials.^13,27,28^ As a typical ceramic material, hexagonal boron nitride (h-BN) has attracted much attention due to its good heat transfer performance, and a large surface area, which provides more active sites for the adsorption process.^29^ Furthermore, the application of BN material in the adsorption process is favored by its two-dimensional layered honeycomb structure, its porosity, hydrophobicity, numerous density defects, good oxidation resistance, good chemical inertness, and smelling capacity.^30–32^ However, the BN is sometimes modified by other materials to enhance its efficiency.^22,33^ Among these materials, graphene is one of the most used materials in the literature to enhance the physicochemical properties of BN.^34–36^

Recently, some works have been carried out to remove MO pollutants from synthetic textile water by the adsorption process. Alghamdi et al.^4^ provided the removal of MO by the adsorption process using KOH polypyrrole-based graphene oxide as an adsorbent. They obtained an MO removal efficiency of 73%. MO removal efficiency of 90% has been found by Asseng et al.^37^ by using activated carbon. In addition, Kamdod et al.^38^ studied the efficiency of tamarind seed-activated carbon modified by chitosan for MO recovery efficiency. In this work, the authors achieved an efficiency of 80%. The synthesis of manganese-based Mn@ZIF-8 nanocomposite by the solvothermal route for MO recovery efficiency was carried out by Nazir et al.^39^ High MO efficiency (91.71%) was achieved. Up to today, researchers have been looking for new adsorbent materials that will show high efficiency in the adsorption process to remove organic pollutants. Thus, GBMs and boron nitride could be very interesting in this field. This is the reason that a low content of graphene has been employed to modify boron nitride and show the efficiency of the as-prepared nanocomposite for MO adsorption. Furthermore, no work using boron nitride- and graphene-based nanocomposites has been found in the literature for MO adsorption.

Within this purpose, we systematically studied the efficiency of graphene-decorated boron nitride (GBN) material for MO removal using a response surface methodology (RSM). Thus, the effect of linear, quadratic, and interaction of the main factors (pH, MO initial concentration, and mass of the adsorbent) was studied. The GBN adsorbent was characterized by a wide range of experimental procedures. Our findings demonstrate that the GBN reaches an efficiency of 99.51% for MO removal, which demonstrates the potential of the proposed material for future technological applications. The results highlight significant reductions in carbon footprint by removing organic pollutants from water compared with conventional activated carbons. Thus, the findings could interest researchers and industries focused on water technology, materials synthesis, and water depollution.

## 2 Experimental Procedure

### 2.1 Chemicals

In this research work, analytical grade chemicals were used without further purification. Hydrochloric acid (HCl, 36.5%), sodium hydroxide (NaOH), and graphene nanoplatelets were purchased from Sigma-Aldrich. MO dye (C_14_H_14_N_3_O_3_SNa, M = 327.33 g/mol, λ_max_= 465 nm, 99.9%) was purchased from Merck.

### 2.2 Preparation of the Adsorbent GBN

A facile preparation route of graphene-decorated boron nitride was employed in this work, and the main steps are summarized in Figure 1. First, 2.5 g of graphene is dispersed in 20 mL of deionized water (DI) and sonicated for 30 min. In another volumetric flask, 2.5 g of boron nitride was dispersed in 20 mL of DI and sonicated for 30 min. Then, both solutions were mixed and stirred for 3 h. After 2 h of agitation, 1 mL of ethylene glycol and 0.05 g of urea were added to the mixture. Afterward, the mixture was washed twice with deionized water and then dried for 12 h in a furnace at 105 °C. The dried material was treated thermally in a tubular furnace at 800 °C for 2 h at a temperature ramp of 5 °C/min in the presence of argon gas. The as-prepared material is named GBN 11. GBN 12 and GBN 13 were also prepared with the same method, with the only difference in the quantity of graphene, which was half and one-third of the boron nitride.

**Figure 1:**
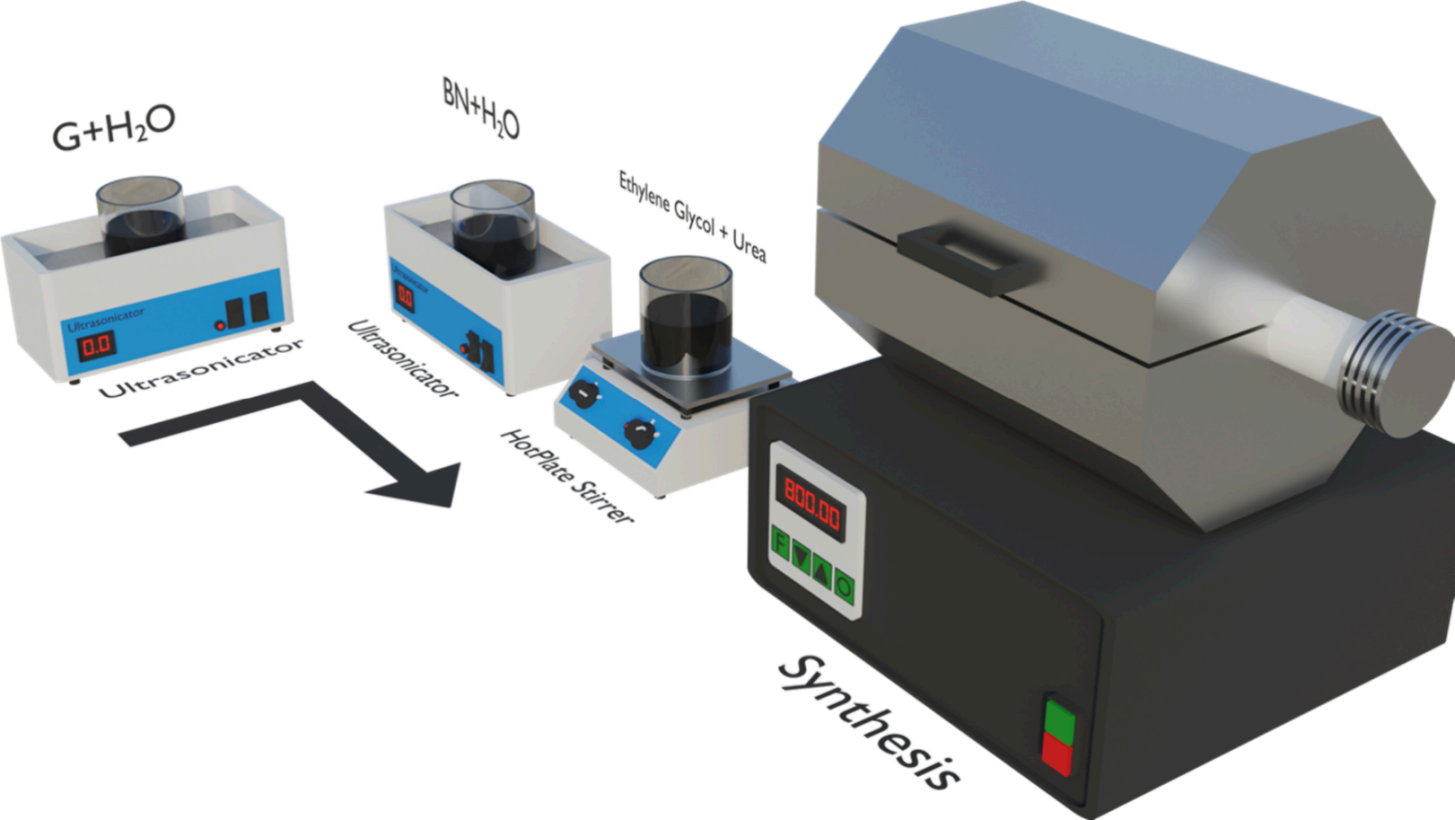
Schematic representation of the synthesis process used to produce the graphene-decorated boron nitride (GBN) material. In the first two steps, an ultrasonicator is used. In the following, a Hot plate stirrer is employed to mix the material. The synthesis is realized using a tubular furnace.

### 2.3 Physicochemical Characterization of the Materials

The X-ray diffraction technique using Rigaku MiniFlex II equipment was employed in this work to examine the crystalline structure of the materials. A field emission scanning electron microscope equipped with energy dispersive X-ray spectroscopy (FEG-SEM Zeiss Supra 35-Vp) was used to characterize the surface morphology, element mapping, and chemical composition of the prepared materials. The determination of functional groups on the surfaces of the materials was carried out by Fourier transform infrared spectroscopy (FTIR, Bruker Tensor 27). The determination of the specific surface area of the as-prepared samples was carried out using an automatic physicochemical isothermal adsorption instrument (BELSORP Mini-II instrument) based on the Brunauer, Emmet, and Teller method. The thermal stability of the material was evaluated by thermogravimetric analysis (TG) coupled to differential thermal analysis (DTA) using a DTG-60H instrument with simultaneous DTA-TG analysis. The UV–vis spectrophotometer (Shimadzu UV-2600 instrument) was used to evaluate the residual MO concentration in aqueous solution at an absorption wavelength of 465 nm.

### 2.4 Adsorption Process

In order to perform the adsorption tests, a standard stock solution of MO dye with an initial concentration of 250 mg/L was prepared and stored in the dark. Each adsorption experiment was carried out with a solution at a determined concentration (20–100 mg/L) obtained by diluting the initial solution and mixing with an adsorbent mass (20–80 mg). The mixture was stirred constantly for a determined time at room temperature (25 °C). Subsequently, the solution was filtered, and the remaining concentration of MO in the solution was evaluated by measuring the absorbance using the UV–visible technique. Before setting up the experimental design, we studied the effects of contact time and initial concentration of MO, which are important factors during the adsorption process. The efficiency of the adsorption process was evaluated from the calculation of the adsorption capacity at time *t* (*q_t_*) given in eq 1. The MO adsorption efficiency (*R*) is calculated according to the following formula (eq 2).^7,40^

$$q_{t}(\mathrm{mg}/g)=\frac{(C_{0}-C_{t})\times V}{m}$$ 1

$$R(\%)=\frac{\left(C_{0}-C_{f}\right)}{C_{0}}\times 100$$ 2

Where *C*_0_ (mg/L) is the initial concentration of the pollutant, *C*_*t*_ (mg/L) is the concentration at time *t*, *V* is the volume of the solution, *m* (g) is the mass of the adsorbent, and *C*_f_ (mg/L) is the final concentration.

### 2.5 Optimization Process

The central composite design (CCD) based on the RSM was used in this research work for modeling and research on the influence of experimental parameters on the response. The experimental design was carried out using Minitab 18 software. CCD is particularly widely used as an experimental design due to its ability to minimize experiments and predict the effects of parameters, individually and in combination.^41^ Three operating parameters, such as adsorbent mass (X_1_), pH (X_2_), and initial dye concentration (X_3_), were used to obtain an experimental matrix design. The experimental domain is presented in Table 1. An analysis of variance (ANOVA) is a widely used statistical tool to show the significance, validation, and adequacy of the postulated mathematical model.^42^ The quadratic polynomial model is given by the following equation.

$$Y=\beta_{0}+\sum \beta_{i}X_{i}+\sum \beta_{\mathrm{ii}}{X_{i}}^{2}+\sum \beta_{\mathrm{ij}}X_{i}X_{j}$$ 3

Where Y is the predicted response, β_i_, β_ii_, and β_ij_ are the linear coefficient, quadratic coefficient, and interaction coefficient of the variables, respectively, and X is the coded factor.

**Table 1:** Experimental Domain

**factors**	**coded factors**		
	**–1**	**0**	**1**
mass: X_1_ (g)	0.02	0.05	0.08
pH: X_2_	2	6	10
initial MO concentration: X_3_ (mg/L)	20	60	100

### 2.6 Regeneration Test of the Adsorbent

The regeneration of the adsorbent after the adsorption process is particularly important in engineering processes and for further application of this process on an industrial scale. In this work, the regeneration of the adsorbent was carried out by the electrochemical AOP using a carbon felt (30 mm, φ = 6 mm) as low-cost electrodes. For this purpose, the dried saturated adsorbent (0.20 g) was put in contact with 100 mL of Na_2_SO_4_ (1.25 g/L) as the supporting electrolyte under stirring for 2 h. After stirring, the solution was filtered, and the filtrate was used to evaluate the release of MO concentration after the regeneration process.^13^ In order to study the number of reuse cycles of the adsorbent material, the test was repeated 10 times.

## 3 Results and Discussion

### 3.1 Comparison of the MO Efficiency Removal on Different Adsorbents

In this research, the comparison of MO adsorption performance on different adsorbents was conducted using the following experimental conditions: adsorbent mass of 20 mg, temperature of 25 °C, initial MO concentration of 100 mg/L, pH of 2, and time of 20 min. Figure 2 presents the MO removal efficiency (%) as a function of the adsorbent. The results indicate that the GBN 11 material has the highest MO removal efficiency, followed by graphene and GBN 12 materials. The GBN 11 adsorbent achieves a removal of 99.21%, while a value around 75.26% is recorded for GBN 12. Meanwhile, graphene attained an efficiency of approximately 85.56%. All of the mentioned materials demonstrate better efficiency compared to the BN materials, which showed values of roughly 22.15%. The findings suggest that graphene significantly enhances the adsorption performance of BN. This could be attributed to the increase in the specific surface area of graphene; however, some relevant research on this topic has indicated that graphene possesses a favorable specific surface area, which enhances its performance in adsorption. Therefore, based on the results obtained with the GBN 11 sorbent, we will concentrate our efforts on the physicochemical characterization of this material to provide a clearer understanding of the adsorption mechanism of MO on this sorbent.

**Figure 2:**
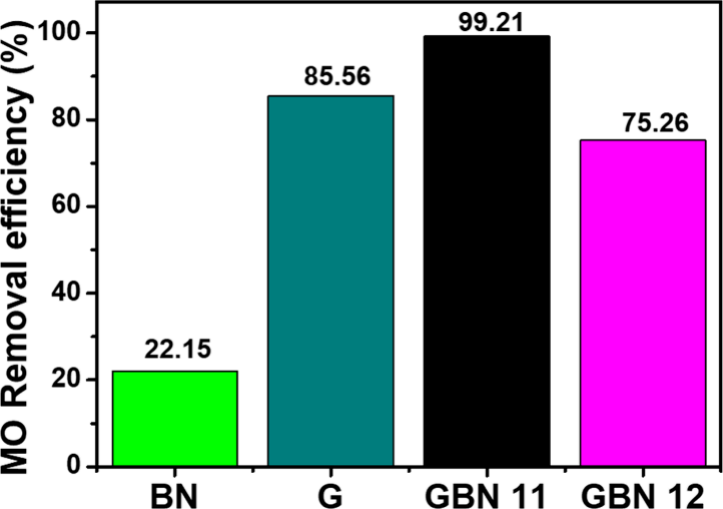
Comparison of the MO removal efficiency of different adsorbents (*m* = 20 mg, temperature = 25 °C, [MO] = 100 mg/L, pH = 2, and time = 20 min).

### 3.2 Physicochemical Characterizations

The morphology and chemical composition of the GBN 11 nanomaterial were determined by using scanning electron microscopy coupled with energy dispersive X-rays (SEM-EDS), with the results presented in Figure 3. Figure 3a displays SEM images of the GBN material, revealing a mixture of lamellar and platelet structures with a rough surface. This structure exhibits stacking and varying thicknesses. Additionally, it is important to note that the GBN 11 sorbent has a high porosity; this may be attributed to the presence of hierarchical boron nitride and graphene nanoplatelets in the prepared material. The results from energy dispersive X-ray spectroscopy (Figure 3b) indicate that the GBN material primarily consists of carbon, boron, nitrogen, and oxygen in the following percentages: 45.51, 44.25, 9.73, and 0.52%, respectively. The presence of nanoplatelets in the nanocomposite structure confirms the modification of the boron nitride structure, which is lamellar. Furthermore, the nearly identical content of carbon and boron demonstrates the addition of carbon to the boron nitride network during the synthesis of the GBN 11 adsorbent. The notable increase in the roughness and porosity of the nanomaterial enhances the adsorption efficiency of the GBN 11 adsorbent. The element distribution in the material is illustrated in Figure 3d–g. From the results, a distribution of elements is observed in the agglomerated material. The carbon and boron contents are nearly similar, indicating the success of the synthesis. Additionally, it is noteworthy that the carbon atom in graphene replaces the nitrogen atoms; when C replaces N, the formed C–B bonds are strong and help preserve the planar hexagonal structure. This may also be due to C (which is less electronegative) replacing N (which is more electronegative), leading to a balanced charge distribution in the lattice and stabilizing the local dipole. Furthermore, during the synthesis of the material, the nitrogen content diminished because nitrogen atoms from BN could incorporate into the graphene matrix, forming nitrogen-doped graphene. Then, at the temperature range of 600–900 °C, the as-formed functional groups (N-graphitic, N-pyrrolic, or N-pyridinic) could be decomposed to release N_2_ or NO_2_.^43^

**Figure 3:**
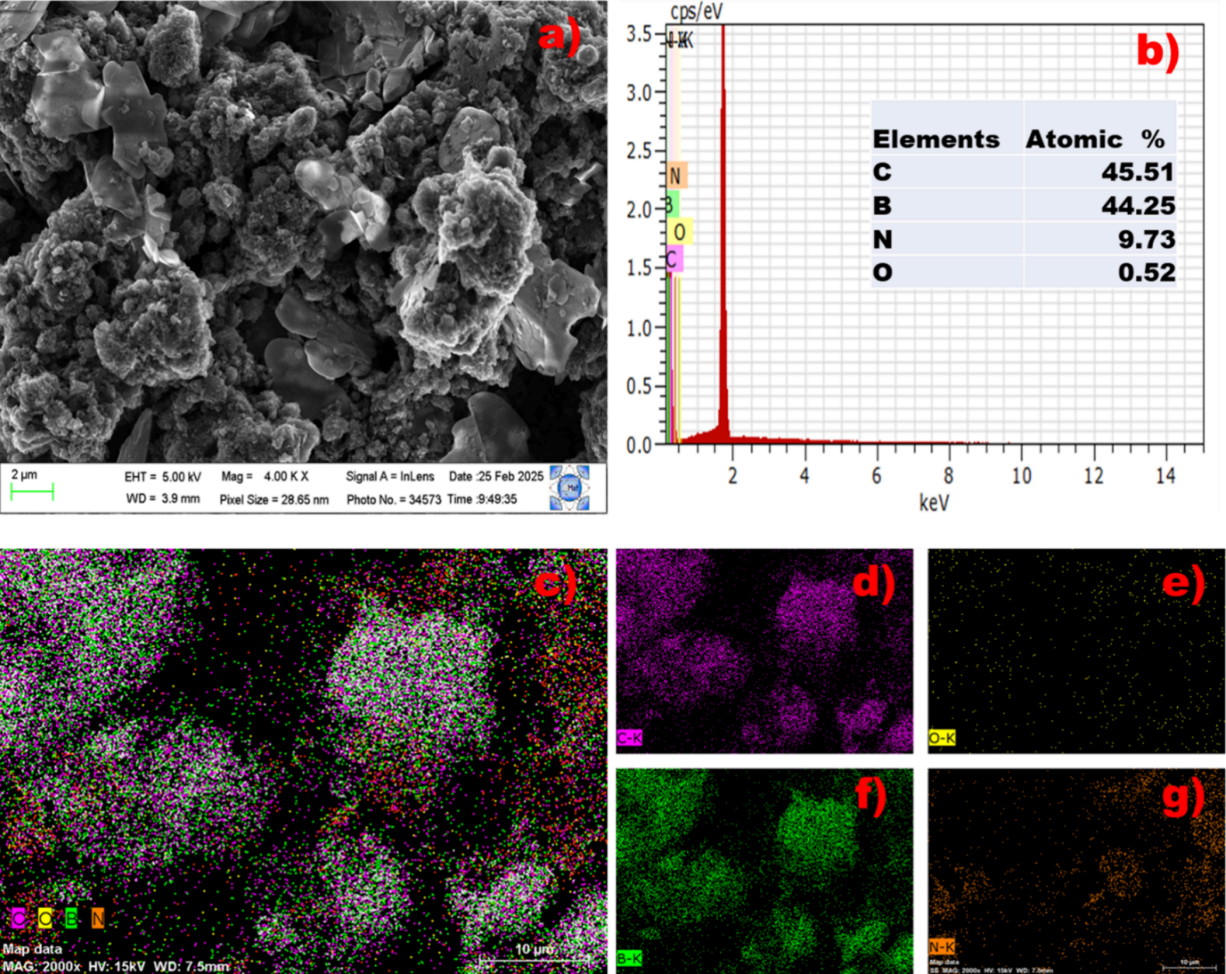
(a) Scanning electron microscopy of GBN 11. (b) EDS spectrum of GBN 11. (c) Distribution of the elements. (d–g) Elemental composition of C, O, B, and N, respectively.

The thermal stability of the GBN 11 material was studied using thermogravimetric analysis (TGA) and DTA. As shown in Figure 4a, the GBN material exhibited an initial mass loss of 3.35% between 35 and 110 °C, which can be attributed to the evaporation of adsorbed water molecules during heat treatment. The second mass loss, which was not significant (2.01%), occurred between 110 and 450 °C and may be due to the removal of hydroxyl groups through dihydroxylation. The final mass loss is substantial (28.57%), occurring between 480 and 900 °C. In this temperature range, the degradation of organic functions present in the GBN 11 nanomaterial during synthesis can be observed. After 900 °C, the material showed no weight loss and is therefore considered to be stable. DTA of the GBN 11 nanomaterial revealed two exothermic peaks at approximately 50 and 880 °C. The first peak relates to dehydration, while the second may be attributed to oxidation of the nanocomposite due to the presence of oxygen atoms from the solvent. However, it is important to note that this peak is broad, which may indicate slow and progressive oxidation during the sorbent synthesis process.

**Figure 4:**
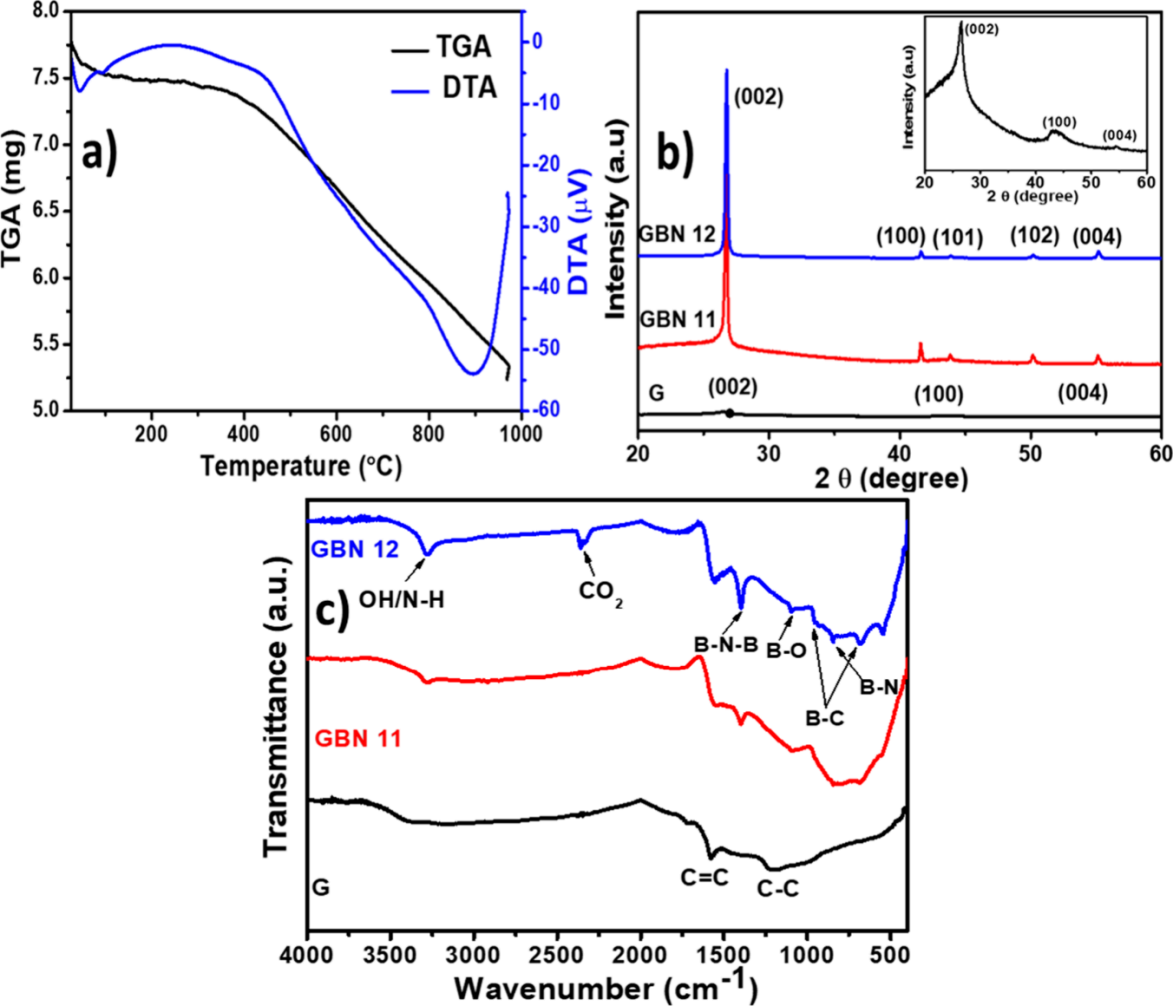
(a) TGA/DTA analyses of the GBN 11 material. (b) XRD patterns of Graphene, GBN 11, and GBN 12 samples. To turn the visualization better, the inset depicts the XRD pattern of graphene, and (c) FTIR spectra of Graphene, GBN 11, and GBN 12 samples.

The X-ray diffractograms of the prepared materials are shown in Figure 4b. The XRD pattern of graphene exhibited three peaks observed at 2θ = 26.2, 43.12, and 54.9° attributed to the graphitic crystal lattices (002), (100), and (004), respectively (JCPDS No. 04-0783).^44^ The XRD pattern of graphene-decorated boron nitride showed the presence of five main peaks. These XRD peaks corresponding to a diffraction angle of 2θ = 26.2, 41.15, 43.12, 49.4, and 54.9° and are indexed to the (002), (100), (101), (102), and (004) lattice planes of h-BN (JCPDS No. 34-0421), respectively.^45–47^ The intensity of the (002) peak increases with the boron nitride content, indicating the good crystallinity of the adsorbent. The diffractogram of the graphene-decorated BN material exhibits peaks similar to those of graphene. This is because both materials (h-BN and graphene) demonstrate a similar crystalline structure (hexagonal) and are 2D crystalline materials. Thus, based on these structural characterization results, it can be concluded that graphene decorated the BN surface and the GBN 11 adsorbent was successfully prepared.

Figure 4c presents the FTIR spectra of a graphene-decorated boron nitride nanomaterial. In the FTIR spectrum of graphene, we can notice the presence of absorption peaks located at 1100 and 1615 cm^–1^, which are attributed to the stretching vibration of C–C (saturated hydrocarbons) and C=C (aromatic chain). In the spectra of the new materials, the presence of new peaks at around 790 and 1385 cm^–1^ is observed, corresponding to the bending vibration of B–N–B (in-plane) and the stretching vibration bands of B–N (out-of-plane), respectively.^48–50^ In the region 3250–3500 cm^–1^, a broad peak is observed and could be attributed to the stretching vibration mode of B-NH_2_/B–OH bonds.^51,52^ The peaks appearing at around 715 and 1075 cm^–1^ could be assigned to the B–C function from the icosahedral boron carbide vibration.^53^ The peak found at 1220 cm^–1^ was attributed to the stretching vibration of the B–O bond. The peak observed at around 2300 cm^–1^ could be assigned to the stretching vibration of the CO_2_ function.^54^ This peak was not observed in the FTIR spectrum of graphene. Its formation could appear during the synthesis process and is further supported by the presence of oxygen, confirmed by the chemical composition of the material. These results prove that the chemical state of the GBN surface has changed and that the presence of these functional groups will contribute to the increase in the adsorption capacity of the material. Especially, the abundance of O–H/N–H functions on the GBN surface could improve its wettability and electronegativity, which improves the rapid adsorption of molecule dyes in aqueous medium.^32^

The specific surface area and pore distribution of the GBN 11 nanomaterial were evaluated by using the BET and BJH methods. Figure 5a presents the N_2_ adsorption–desorption isotherms as a function of the relative pressure. This figure shows a general shape characteristic of type I isotherms with a type of H_2_ hysteresis loop. The presence of both types of isotherms in the material indicates that the sorbent contains both mesopores and micropores. The increase in N_2_ adsorption at low relative pressure may be attributed to the filling of the material’s micropores, thus confirming the presence of type I. The mesoporous structure of the material is supported by the hysteresis observed in the adsorption–desorption curve. Figure 5b presents the pore size distribution of the GBN 11 material. This figure clearly shows the existence of micropores (*d* < 2 nm) and mesopores (2 nm < *d* < 50 nm). However, the micropores in a material play a fundamental role in enhancing the π–π interactions between the adsorbate and the adsorbent, leading to increased process efficiency. Moreover, the adsorption performance of the GBN 11 material could also be improved due to the presence of mesopores, which facilitate the diffusion and mass transfer of the adsorbate on its surface. The BET surface area, total pore volume, and average pore diameter of the GBN 11 adsorbent were 461.6 m^2^/g, 0.47 cm^3^/g, and 4.075 nm, respectively. On the other hand, graphene has a BET surface area of 383.4 m^2^/g, a total pore volume of 0.295 cm^3^/g, and an average pore diameter of 4.041 nm. The GBN 11 adsorbent exhibited a high surface area compared to graphene. Furthermore, the important pore volume of GBN 11 indicates its ability to adsorb MO molecules and enhance the adsorption process.

**Figure 5:**
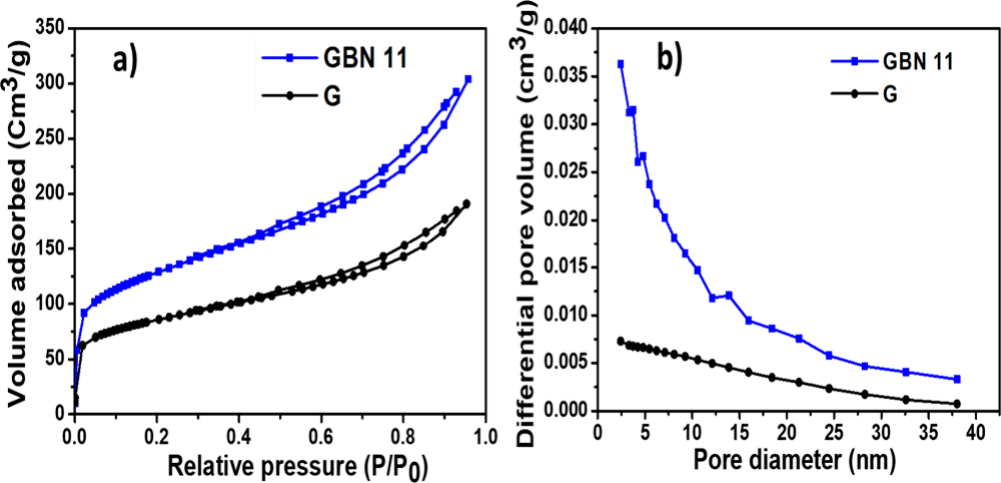
(a) N_2_ adsorption–desorption isotherms. (b) Pore diameter distribution of GBN 11.

### 3.3 Optimization of the MO Removal Rate

The optimization of the MO removal efficiency using the GBN 11 adsorbent was carried out the RSM. CCD and RSM were employed to determine the impact of operating factors on the MO removal rate in a synthetic solution. Thus, three main factors (mass of adsorbent, pH, and initial MO concentration) were selected due to their influence on the response during the adsorption process. The results are shown in Table 2. The CCD was designed with three levels and six center points to minimize errors during the experiments. Consequently, 20 experiments were designed, analyzed, and adjusted based on ANOVA analysis. The MO adsorption efficiency ranges from 87.34 to 98.90%. The lowest MO removal efficiency value (87.32%) was obtained under the following conditions: X_1_ = 0.02 mg, X_2_ = 10, and X_3_ = 100 mg/L. Conversely, the highest MO removal efficiency (98.905%) was achieved by considering X_1_ = 0.08 mg, X_2_ = 2, and X_3_ = 100 mg/L. Based on these results, the postulated mathematical model (coded factors) is obtained and presented in eq 4. The coefficients of each factor indicating their influence on the response are shown in Table S1 of the Supporting Information. It is important to note that a negative coefficient in the equation indicates an antagonistic effect, while a positive coefficient indicates a synergistic effect on the MO adsorption efficiency.

**Table 2:** Experimental Matrix with Experimental and Predicted Responses

	factors			adsorption yield (%)	
run	X_1_ (g)	X_2_	X_3_ (mg/L)	exp.	pred.
1	0.08 (1)	10 (1)	20 (−1)	93.621	93.747
2	0.02 (−1)	2 (−1)	20 (−1)	95.683	96.382
3	0.08 (1)	10 (1)	100 (1)	97.848	97.010
4	0.08 (1)	2 (−1)	20 (−1)	95.554	94.535
5	0.05 (0)	6 (0)	60 (0)	96.327	96.439
6	0.05 (0)	6 (0)	60 (0)	96.134	96.439
7	0.02 (−1)	10 (1)	20 (−1)	93.621	93.146
8	0.05 (0)	6 (0)	60 (0)	96.413	96.439
9	0.02 (−1)	10 (1)	100 (1)	87.320	88.199
10	0.05 (0)	6 (0)	60 (0)	96.327	96.440
11	0.02 (−1)	2 (−1)	100 (1)	93.144	92.879
12	0.08 (1)	2 (−1)	100 (1)	98.905	99.241
13	0.05 (0)	6 (0)	20 (−1)	93.551	94.222
14	0.08 (1)	6 (0)	60 (0)	90.961	92.356
15	0.05 (0)	10 (1)	60 (0)	95.404	95.712
16	0.05 (0)	6 (0)	100 (1)	94.214	94.102
17	0.05 (0)	2 (−1)	60 (0)	98.196	98.446
18	0.05 (0)	6 (0)	60 (0)	96.392	94.634
19	0.05 (0)	6 (0)	60 (0)	94.550	94.634
20	0.02 (−1)	6 (0)	60 (0)	89.712	88.874

#### 3.3.1 Postulated Mathematical Model and Model Validation

$$Y=93.90+371.4X_{1}-2.295X_{2}-0.0380X_{3}-4466{X_{1}}^{2}+0.1528{X_{2}}^{2}-0.000296{X_{3}}^{2}+5.10X_{1}X_{2}+1.710X_{1}X_{3}-0.00226X_{2}X_{3}$$ 4

The application of the statistical analysis based on ANOVA for the MO removal in the synthetic solution is depicted in Table 3. Since the P-value and F-value are mainly used to show the significance of one factor during the experimental design, they were employed in this study. From Table 3, it is observed that the p-value was <0.001, and the F-value was 13.15 > 5. These results demonstrate the significance of the established model. On the one hand, the significant model factors were X_1_, X_2_, X_1,_^2^ X_2,_^2^ and X_1_X_3_. On the other hand, the nonsignificant model factors were X_3_, X_3_^2^_,_ X_1_X_2_, and X_2_X_3_.

**Table 3:** Analyses of Variance

	**DF**	**adj SS**	**adj MS**	***F*-value**	***P*-value**
MO model	10	144.895	14.489	13.15	0.000
blocks	1	12.091	12.092	10.98	0.009
linear	3	49.023	16.341	14.84	0.001
X_1_	1	30.304	30.304	27.51	0.001
X_2_	1	18.682	18.683	16.96	0.003
X_3_	1	0.036	0.036	0.03	0.860
square	3	53.788	17.929	16.96	0.003
X_1_^2^	1	43.360	43.360	39.37	0.000
X_2_^2^	1	16.037	16.038	14.56	0.004
X_3_^2^	1	0.600	0.600	0.54	0.479
2-way interaction	3	37.731	12.578	11.42	0.002
X_1_X_2_	1	2.997	2.998	2.72	0.133
X_1_X_3_	1	33.692	33.692	30.59	0.000
X_2_X_3_	1	1.042	1.042	0.95	0.356
error	9	9.913	1.102		
lack of fit	5	8.176	1.635	3.76	0.112
pure error	4	1.737	0.434		
total	19	154.808			
*R* ^2^ = 93.60%			adj *R* ^2^= 86.46%		

This observation is confirmed by the low P-value and high F-value presented in Table 3. Furthermore, it is observed that the MO removal efficiency is greatly influenced by the mass of the adsorbent and slightly influenced by the initial MO concentration. Additionally, the postulated model shows a lack of fit, with F and P values of 3.76 and 0.112, respectively. This result indicates that the lack of fit is not significant, confirming the alignment of the experimental and predicted data. Moreover, the high R^2^ value (93.60%) and adjusted R^2^ (86.46%) for the established mathematical model suggest that the proposed model fits well with the experimental data and can effectively elucidate the optimization process.

A Pareto graph was plotted and presented in Figure S1 (see Supporting Information) to further analyze the linear, quadratic, and interaction effects of factors on the MO removal efficiency. From this plot, it is noted that the quadratic effect of mass strongly influences the response, followed by the interaction between the adsorbent mass and the initial pollutant concentration. The normal probability curve for the residuals and the predicted versus experimental response curves were used to confirm the results presented by the ANOVA.

#### 3.3.2 2D Contour Plots

As the experimental data fit well with the predicted data, we can use the postulated mathematical model to plot the contour curves. These graphs help us to understand how each independent factor or their interaction influences the response and to determine the optimal values of the factors. Figure 6a presents the contour curve plot of the interaction between mass and pH on the MO removal efficiency. This figure revealed that the highest MO removal efficiency (99.50%) is obtained when combining a pH value (pH = 2) and an adsorbent mass of around 0.062 g. Increasing the pH results in a decrease in the MO removal efficiency. Whereas increasing the adsorbent mass up to 0.062 g increases the response, but decreases above this mass. Thus, to maximize the response of MO removal efficiency, we need to keep the mass of adsorbent around 0.062 g and pH = 2. Since MO is an anionic dye, it is observed that the removal efficiency of MO increases as the pH decreases. This is because by decreasing the pH, the negative charges on the surface of the adsorbent also decrease, and thus lead to a stronger electrostatic interaction between the adsorbent and the MO molecules present in solution.^55^ In some works, it is well reported that the presence of protons or hydroxide ions in the medium significantly influences the charges of the sites on the surface of the adsorbent and the removal efficiency of organic dyes.^5,38^ Thus, the pH is one of the key factors in the adsorption process. Kamdod et al. conducted a study on the removal of MO in aqueous media by an activated carbon obtained from chitosan-modified tamarind seeds.^38^ They showed that a high MO adsorption capacity was obtained at pH 3.

**Figure 6:**
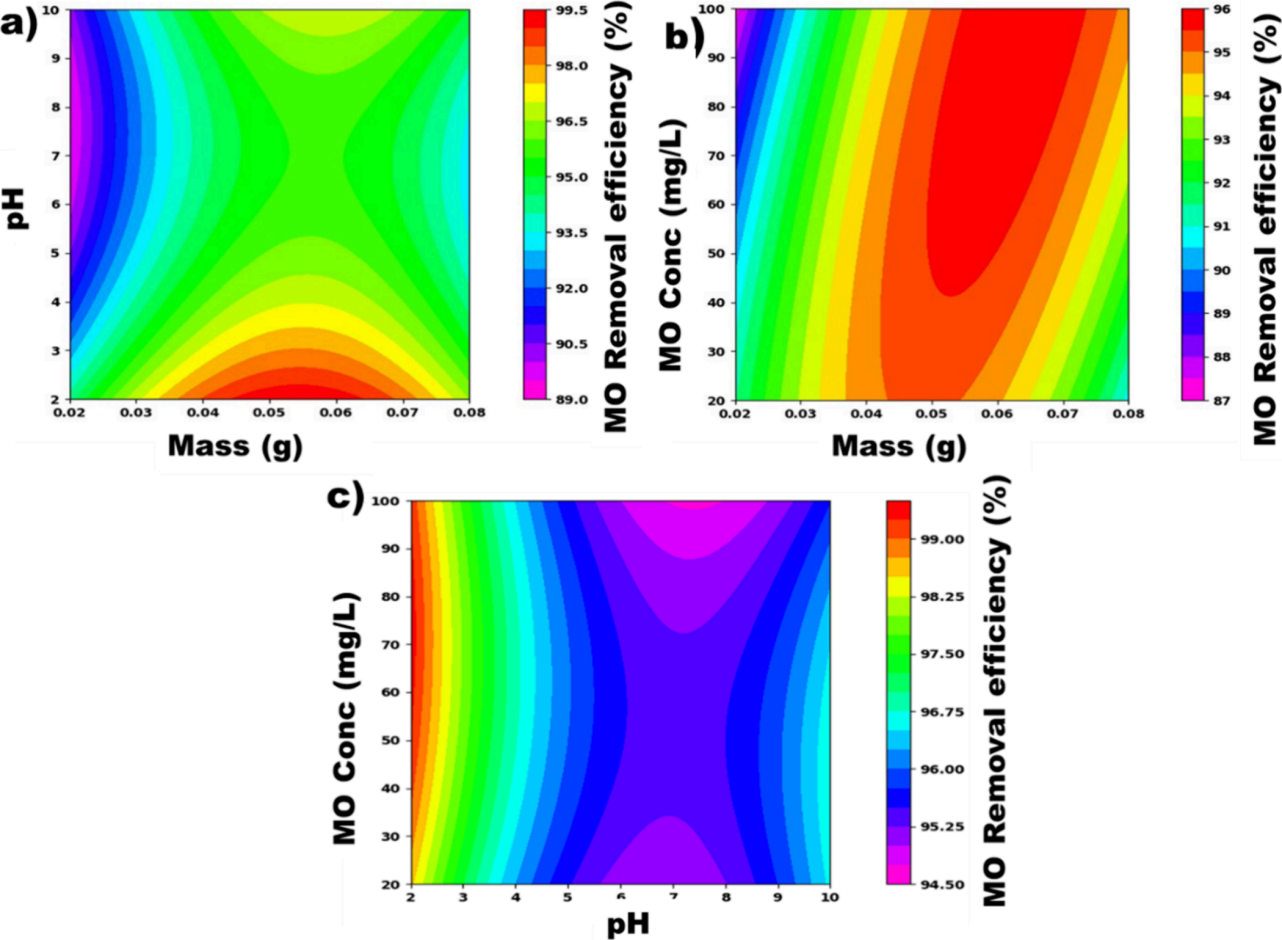
2D contour plots of the interaction between (a) mass and pH. (b) Mass and MO initial concentration. (c) pH and MO initial concentration.

Figure 6b presents the influence of the interaction between adsorbent mass and initial MO concentration on the MO removal efficiency. These results show that the interaction between the adsorbent and the MO concentration strongly influences the MO removal efficiency. However, the MO removal efficiency increases slowly with the adsorbent mass and rapidly with the MO concentration. Moreover, when the adsorbent mass is greater than 0.062 g, a decrease in efficiency is observed. This could be because a high adsorbent mass in the effluent to be treated can cause agglomeration of adsorbent particles, leading to saturation and/or blocking of adsorption sites, followed by a decrease in the efficiency of the adsorption process.^40,56^ On the other hand, the MO removal efficiency increases with the initial MO concentration. Thus, the highest MO removal efficiency is observed at an initial MO concentration of approximately 100 mg/L.

Figure 6c shows the effect of the interaction between pH and the initial MO concentration on the response. This figure shows that a good combination of pH and initial MO concentration around pH = 2 and [MO] = 100 mg/L leads to a high MO removal efficiency (99.19%).

### 3.4 Optimization of MO Removal Efficiency on GBN Adsorbent

The optimal values of the operational factors and the response were obtained with the software by maximizing the responses. Table 4 presents a summary of the optimal values. To achieve maximum MO removal efficiency (99.51%), it is recommended to use an adsorbent mass of 0.062 g, a pH of 2.0, and an initial MO concentration of 100.0 mg/L. However, to determine the actual optimal value, the average of three experiments was conducted using the optimal values of each factor. Compared to other studies on MO removal in aqueous media through the adsorption process, the results obtained in this study are noteworthy because a high MO concentration was used, a short processing time, and a low adsorbent mass resulted in high MO removal efficiency. Table 5 summarizes several studies on MO removal in aqueous media with different adsorbents and their main results. The good efficiency of the GBN 11 sorbent could be attributed to the high specific surface area and facile recombination rate of the GBN 11 nanocomposite. Our findings were also reported in previous studies using BN or graphene as an adsorbent.^23,57,58^

**Table 4:** Validation and Optimization

**factors**	**optimal values**	**predicted optimal MO removal efficiency (%)**	**real optimal MO removal efficiency (%)**
mass: X_1_ (mg)	0.062	99.81	99.51
pH: (X_2_)	2.000		
MO concentration: X_3_ (mg/L)	100.000		

**Table 5:** Comparison of MO Removal Efficiency by Various Adsorbents

adsorbent	mass (g)	time (min)	efficiency (%)	*q*_m_ (mg/g)	references
magnetic activated carbon	0.04	60	96.29	218.00	^59^
tamarind seed-activated carbon	0.01	80	95.56	94.45	^38^
activated biocarbon	0.12	1020	98.12	220.19	^7^
Ca(OH)_2_/Na_2_FeO_4_	0.12	40	99.20	140.10	^60^
polyamine/activated carbon	0.025	120	70.00	285.00	^61^
mushroom root-activated carbon	0.02	60	98.77	286.99	^62^
manganese-based Mn@ZIF-8 nanocomposite	0.01	120	91.71	406	^39^
silica gel (Si–NCON_2_H_3_)	0.01	60	89.15	133	^19^
GBN	0.062	20	99.51	322.52	this work

### 3.5 Adsorption Kinetics

The adsorption kinetic results are presented in Figure 7a. This figure shows that the adsorption process is rapid during the first few minutes and then stabilizes after 20 min. This confirms the presence of a strong interaction between the pollutant and the GBN 11 adsorbent present in the effluent. Maximum MO removal efficiency is achieved after 20 min of processing.

**Figure 7:**
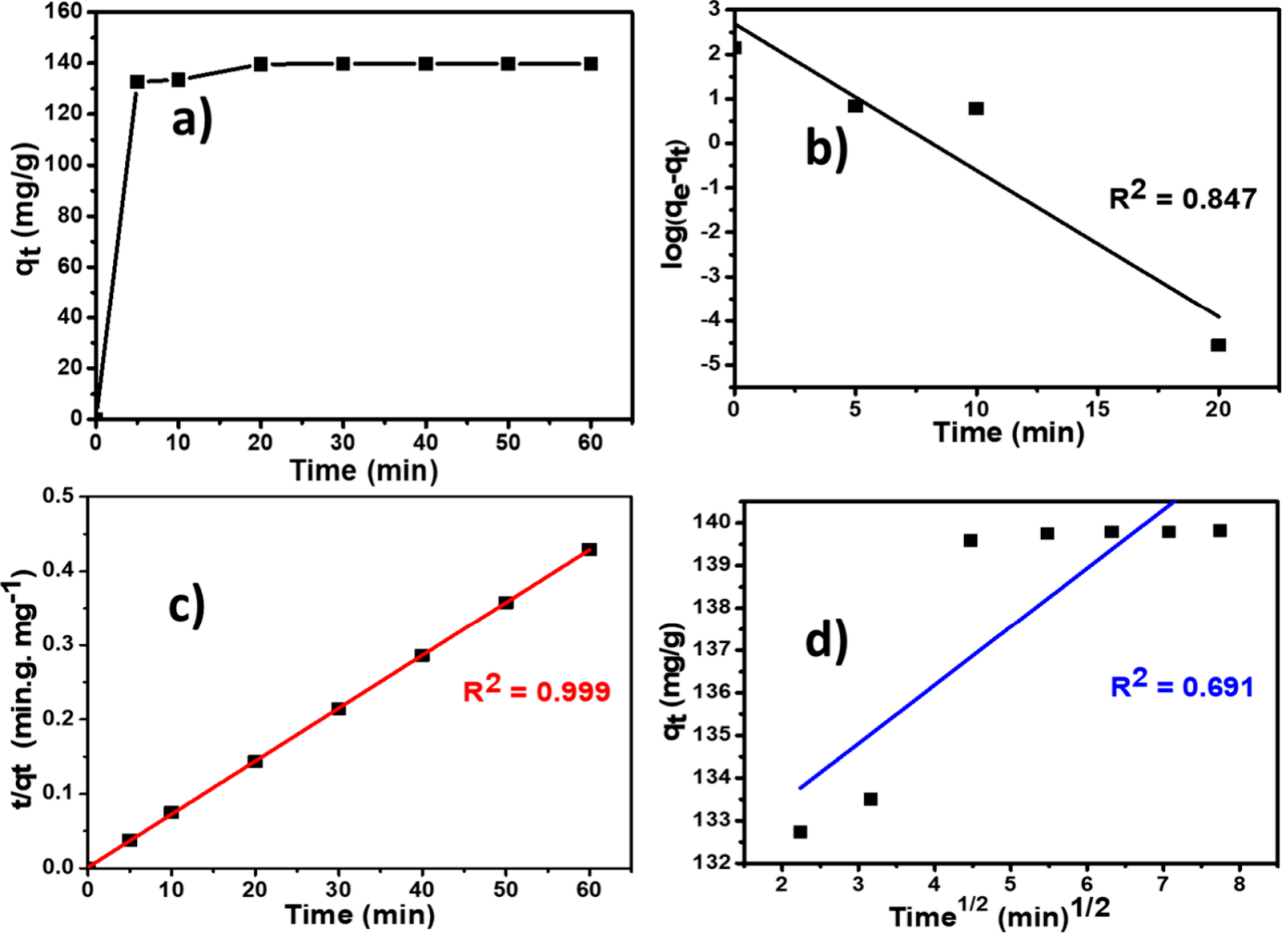
(a) Kinetic study. (b) Pseudo-first-order model. (c) Pseudo-second-order model. (d) intraparticle diffusion model (*m* = 0.062 g, temperature = 25 °C, [MO] = 100 mg/L, and pH = 2).

In this work, pseudo-first-order (eq 5), pseudo-second-order (eq 6), and intraparticle diffusion (eq 7) kinetic models were used to model the adsorption kinetics, and the results are presented in the following curves (Figure 7b–d). The adsorption rate constants of the MO according to the different models are reported in Table S2. From these results, we can notice that the pseudo-second-order kinetic model, which has a higher coefficient of determination *R* ^2^ = 0.999, best describes the adsorption kinetics of MO on the GBN adsorbent. Thus, this result suggests that the chemisorption is the rate-limiting step that controls the adsorption process.^31^ For pseudo-first-order and intraparticle diffusion, the values of the correlation coefficient (*R* ^2^) are relatively low. Thus, the *q*_e_ calculations for these two models show that the theoretical quantities of the pollutants are lower than those of the experimental quantities. Therefore, it can be affirmed that the adsorption process of MO on the GBN material does not occur by a controlled diffusion process but depends on the initial concentration of MO and the contact time.^49,63^

$$\log(q_{e}-q_{t})=\log\;q_{e}-\frac{k_{1}}{2303}t$$ 5

$$\frac{t}{q_{t}}=\frac{1}{K_{2}q_{e}^{2}}+\left(\frac{1}{q_{e}}\right)t$$ 6

$$q_{t}=k_{d}t^{0.5}+C$$ 7

Where, *q*_e_ and *q*_*t*_ are the adsorption capacities (mg/g) at equilibrium and at time *t*, respectively, *k*_1_ (min^–1^), *k*_2_ (g/min mg), and *k*_d_ (mg/g min^0.5^) are the pseudo-first, pseudo-second order, and intraparticle diffusion rate constants, respectively, *t* time (min), and *C* is a constant for the boundary layer thickness.

### 3.6 Adsorption Isotherms

Since the initial concentration of MO is a factor that most influences the adsorption process, its study was carried out in this work, and the result is presented in Figure 8a. We observe in this figure that the adsorption capacity of the MO dye increases with the initial concentration of MO. However, high adsorption capacities are obtained at high concentrations of MO due to the rapid adsorption and saturation of the adsorption sites available on the surface of the adsorbent. This could also be due to the formation of the π=π stacking interactions between the MO molecules and the GBN 11 adsorbent. This finding was also found in previous work.^31^ Subsequently, the adsorption isotherms were studied in this work to evaluate the adsorption capacity and the interaction between the adsorbate (MO) and the adsorbent (GBN = 11). Thus, three different isotherms were used to explain the distribution mechanism of adsorbates on the interface (solid–liquid) at equilibrium by fitting the experimental data. The equations of the different Langmuir (eq 8), Freundlich (eq 9), and Temkin (eq 10) models are presented below.^1,40,64^

$$q_{e}=\frac{q_{m}K_{L}C_{e}}{1+K_{L}C_{e}}$$ 8

$$q_{e}=k_{f}{C_{e}}^{1/n}$$ 9

$$q_{e}=\frac{RT}{B}\ln(K_{r}C_{e})$$ 10

Where *q*_e_ (mg/g) is the adsorbed quantity of the solute at equilibrium, *q*_m_ the maximum adsorption capacity expressed in mg/g, *C*_e_ the concentration of the solute at equilibrium in (mg/L), and *k*_L_ the Langmuir constant expressed in L/mg. *K*_f_ is the Freundlich constant characterizing the effectiveness of an adsorbent for a given solute; *n* is a nonlinear factor reflecting the heterogeneous energy of the adsorption surface. If *n* > 1, then the adsorption is quantitatively greater, and if *n* < 1, the adsorption is weak. *K*_r_ is the Temkin constant (L/mg), and *B* is another Temkin parameter (L/mg).^56,65^

**Figure 8:**
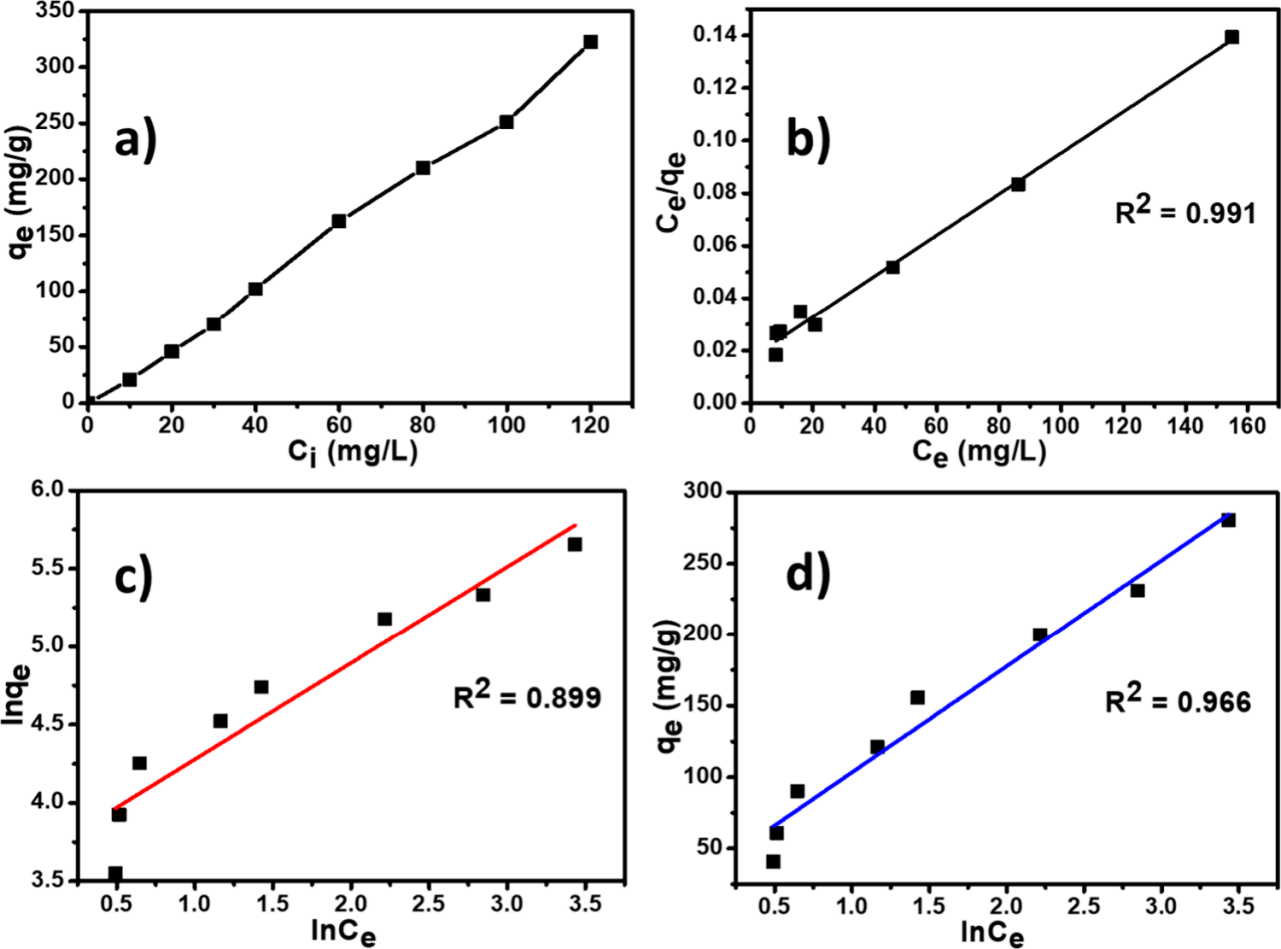
(a) Effect of the MO initial concentration on the MO removal efficiency. (b) Langmuir isotherm. (c) Freundlich isotherm. (d) Temkin isotherm models (*m* = 0.062 g, temperature = 25 °C, *t* = 20 min, and pH = 2).

In the adsorption process, adsorption isotherms are widely used to understand the adsorption mechanism of a pollutant on the adsorbent surface. The models used in this work are the Langmuir (Figure 8b), Freundlich (Figure 8c), and Temkin (Figure 8d) models, which are the simplest and most widespread for this purpose. From the equations of the different models, the data were extrapolated and are presented in Table S3. Regarding the correlation coefficient *R* ^2^, it appears that the Langmuir (*R* ^2^ = 0.9927) and Temkin (*R* ^2^ = 0.9716) isotherms further explain the adsorption mechanism of this study. Thus, the process can be described by the following statements: MO molecules are adsorbed in a single layer without interaction between MO-MO molecules. According to the Temkin isotherm, MO adsorption occurs in multilayers on the GBN surface. These multilayers could be related to the MO interactions with functional groups of the composite.^58^ Finally, the adsorption energy of the MO decreases linearly with the coverage of the adsorbent surface.

### 3.7 Mechanism of MO Sorption on GBN

According to the results of the physicochemical characterization of the adsorbent material, the study of the adsorption kinetics, and the modeling of the adsorption isotherms, we can propose an adsorption mechanism of MO molecules on the surface of our sorbent material (GBN 11). It is well-known that, depending on the surface and textural properties of the adsorbent, both mechanisms (chemical and physical) of sorption could appear during the process.^59^ According to the results of the determination of the functional groups that the adsorbent presents by FTIR, the MO as an anionic dye could be attached to the surface of the GBN material via ion exchange between the positively charged ammonium group and the negatively charged MO molecules. This interaction is shown in Figure 9. In addition, the compact hexagonal crystalline structure of the GBN material could contribute to the formation of π–π interactions with the aromatic ring of MO molecules through electron donor–acceptor interactions and therefore improves its adsorption capacity.^62^ In this figure, the formation of hydrogen bonds between the N atoms of the MO molecules and the H atoms of the hydroxyl (−OH) present on the surface of the adsorbent is also highlighted. In addition, an electrostatic interaction may occur during the adsorption process due to the positive charge of the adsorbent and the negative charge of the MO molecules. According to the BET analysis, GBN has a pore size of 4.075 nm, which is larger than that of the MO molecules (1.44 nm × 1.09 nm × 0.50 nm). This helps to increase pore availability through easy filling and interaction between GBN 11 and the MO molecules. This result could be confirmed by the large specific surface area of the GBN 11 adsorbent.

**Figure 9:**
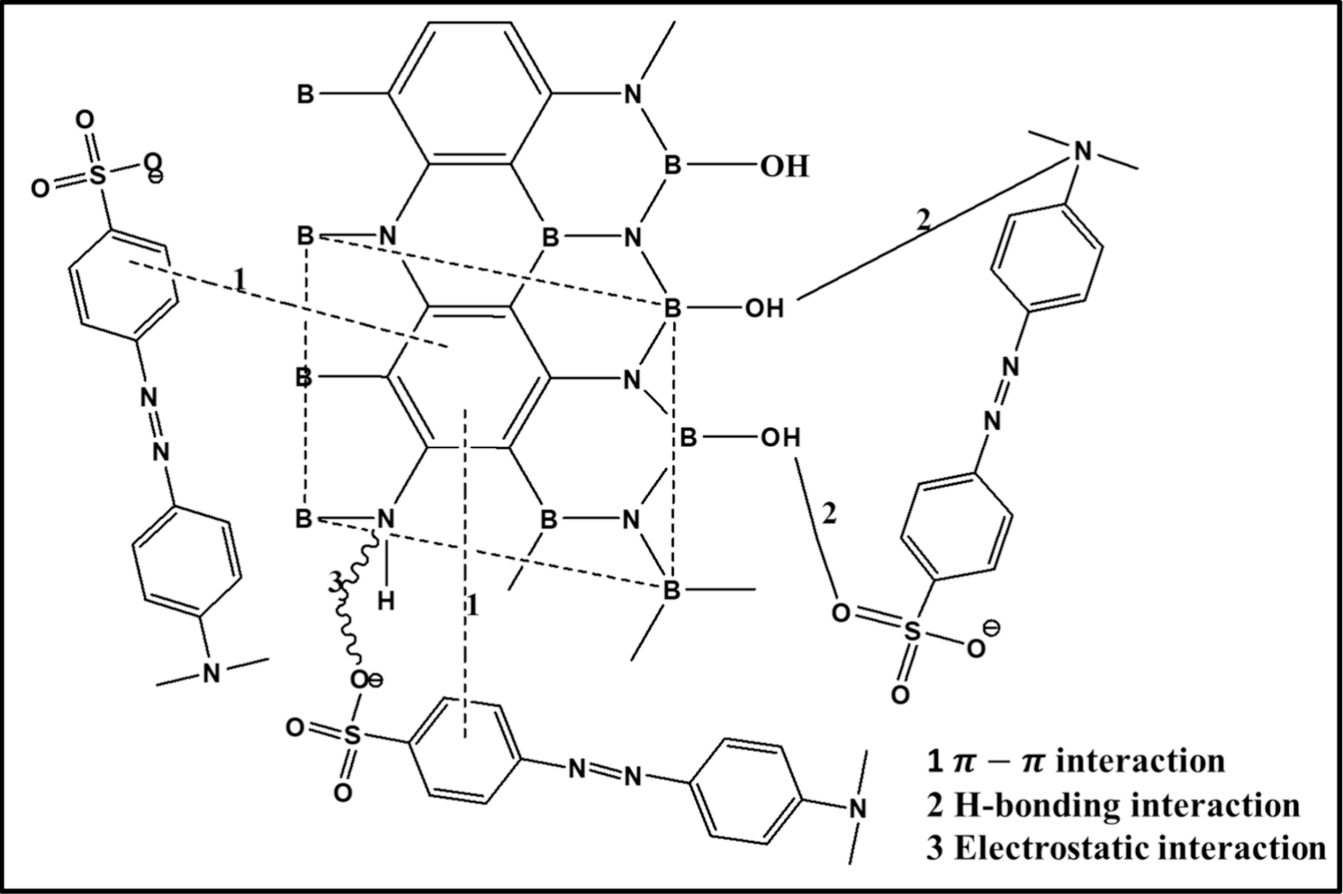
Plausible mechanism of MO ion adsorption onto GBN.

### 3.8 Regeneration Studies

The regeneration of adsorbents is important for their reusability and is a key point for their use on an industrial scale. In this study, the regeneration study of the adsorbent was carried out by a direct anodic oxidation process. In this process, a highly oxidant, powerful species (hydroxyl radicals) is generated in the solution (eq 11) and could oxidize the pollutant up to the mineralization stage. Each cycle was repeated three times, and the average was used to plot the graph of the MO removal efficiency versus the cycles. Thus, the optimal values of the experimental factors obtained from the mathematical model were used in this part. Figure 10 shows that the MO removal efficiency decreases slowly with cycles. The graphene-decorated boron nitride material showed only a negligible decrease in its efficiency of 0.96 and 2.68%, after 5 and 10 cycles of use, respectively. During the regeneration of the GBN 11 adsorbent, the MO removal rate was 100%. This is because the AOP used in this work leads to the electrogeneration of hydroxyl radicals, which have a high oxidation potential (*E* ^0^ = 2.8 V/SHE) and react with MO molecules until their complete degradation.^13^ Furthermore, the good reusability of the nanocomposite could be attributed to the chemical inertness of boron nitride- and graphene-based materials.^66^ Thus, the GBN material could be used several times after regeneration by the direct anodic oxidation process.

$${\mathrm{MO}}_{x}+H_{2}O\to {\mathrm{MO}}_{x}{(}^{\cdot }\mathrm{OH})+H^{+}+e^{-}$$ 11

**Figure 10:**
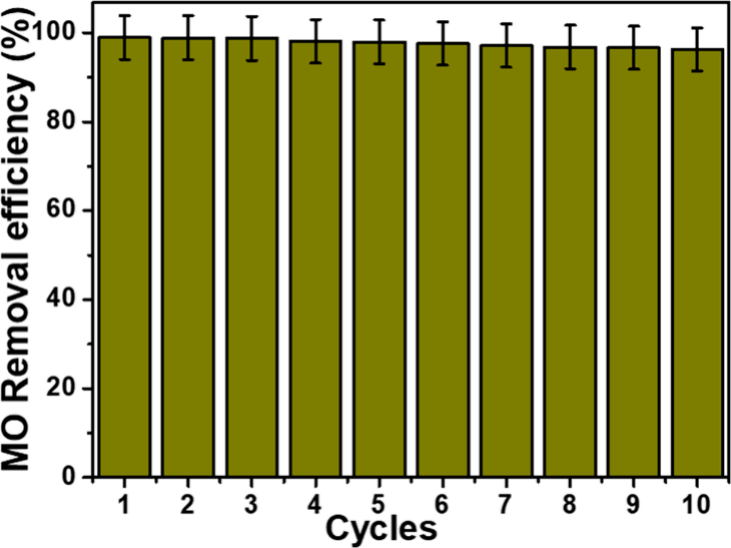
MO removal efficiency of the regenerated GBN 11 adsorbent (*m* = 0.062 g, [MO] = 100 mg/L, temperature = 25 °C, *t* = 20 min, and pH = 2).

## 4 Conclusion

In summary, the physicochemical characterizations of the material confirmed the successful preparation of GBN and the insertion of graphene into the lattice of boron nitride for the removal of organic pollutants in water applications. The GBN 11 material showed the best adsorption performance compared to other adsorbents. The optimization of the MO removal efficiency by GBN led to the determination of the mathematical model and optimal values of the MO adsorption parameters. The total MO removal efficiency was achieved at a contact time of 20 min, a temperature of 25 °C, an adsorbent mass of 0.062 g, an initial MO concentration of 100 mg/L, and a pH of 2. In addition, the pseudo-second-order kinetic model and the Langmuir and Temkin isotherm models were well fitted to the experimental data. According to these results, MO molecules are adsorbed in single and multiple layers, without interaction between them (MO–MO), which leads to an increase in the order of their distribution on the surface of the GBN 11 material. Based on our findings, the as-prepared sorbent can be a powerful candidate for the removal of organic pollutants, especially dyes, in textile wastewater. We go beyond; here, it was demonstrated that the MO removal efficiency remains essentially unchanged after 10 cycles, showing its excellent recyclability.

## Supplementary Material



## Notes

The Supporting Information provides additional information about the MO removal process. More specifically, this document shows Table S1, where the coefficients and the influence of each factor are depicted. Figure S1 shows the standardized Pareto plot parameters and the correlation between the experimental and predicted data. Tables S2 and S3 depict the absorption kinetic parameters and fitting results for isotherm models, respectively (PDF)
