# Supplementary material for: High Efficiency of the Graphene-Decorated Boron Nitride for Dye Removal from Aqueous Solution: Modeling and Optimization Process Designed for Textile Wastewater Treatment
Source: ACS Omega. 2025 Oct 24;10(43):51686–99. doi: 10.1021/acsomega.5c07579 (PMC12593146; doi:10.1021/acsomega.5c07579)
Supplement: Supplementary file 1 [file ao5c07579_si_001.pdf]

High Efficiency of the Graphene Decorated Boron Nitride Used for Dye Removal in Aqueous Solution; Modeling and Optimization Process Designed for Textile Wastewater Treatment

Romuald Teguia Doumbi[1,2*], Paulino Vasco Mariano Muguirrima[1], Artur de Morais[1], Felipe Bohn[2], Carlos Alberto Martínez-Huitle[3], Igor Cretescu[4], Marcio Assolin Correa[1,2,]

[1] Postgraduate Program in Materials Science and Engineering, Federal University of Rio Grande do Norte, Natal, RN, 59078-970, Brazil

[2] Department of Physics, Federal University of Rio Grande do Norte, Natal, RN, 59078-970, Brazil

[3]Renewable Energies and Environmental Sustainability Research Group, Institute of Chemistry, Federal University of Rio Grande do Norte, Natal, RN, 59078-970, Brazil

[4]''Gheorghe Asachi" Technical University of Iasi, ''Cristofor Simionescu" Faculty of Chemical Engineering and Environmental Protection, 73 Blvd, Mangeron, Iasi, 700050, Romania

Table S1 presents the coefficients of each factor, indicating their influence on the MO removal efficiency based on the T-value and P-values. P-value of the postulated model is < 0.05, indicating the significance of the results obtained from the model. The factor that had the highest influence on the response was the mass of the adsorbent (X_1).

Table S1. Coded factors and their coded coefficients

Coded Coefficients				
Term	Coef	SE Coef	T-Value	P-Value
Constant	95.537	0.373	256.22	0
Blocks				
1	0.903	0.272	3.31	0.009
X_1	1.741	0.332	5.25	0.001
X_2	-1.367	0.332	-4.12	0.003
X_3	-0.06	0.332	-0.18	0.86
X_1^2	-4.019	0.641	-6.27	0
X_2^2	2.444	0.641	3.82	0.004
X_3^2	-0.473	0.641	-0.74	0.479
X_1X_2	0.612	0.371	1.65	0.133
X_1X_3	2.052	0.371	5.53	0

X_2X_3	-0.361	0.371	-0.97	0.356

Figure S1 illustrates the Pareto graph, the normal probability distribution, and the correlation between the experimental results and the predicted results. The Pareto graph (Figure S1a) further illustrates the influence of each factor and its combined effect on the response. Figure S1b shows a good distribution of the normal probability. The coefficient of correlation is $R^2 = 0.936$ (Figure S1c). This value indicates that only 6.4% of the results have not been explained by the established model.

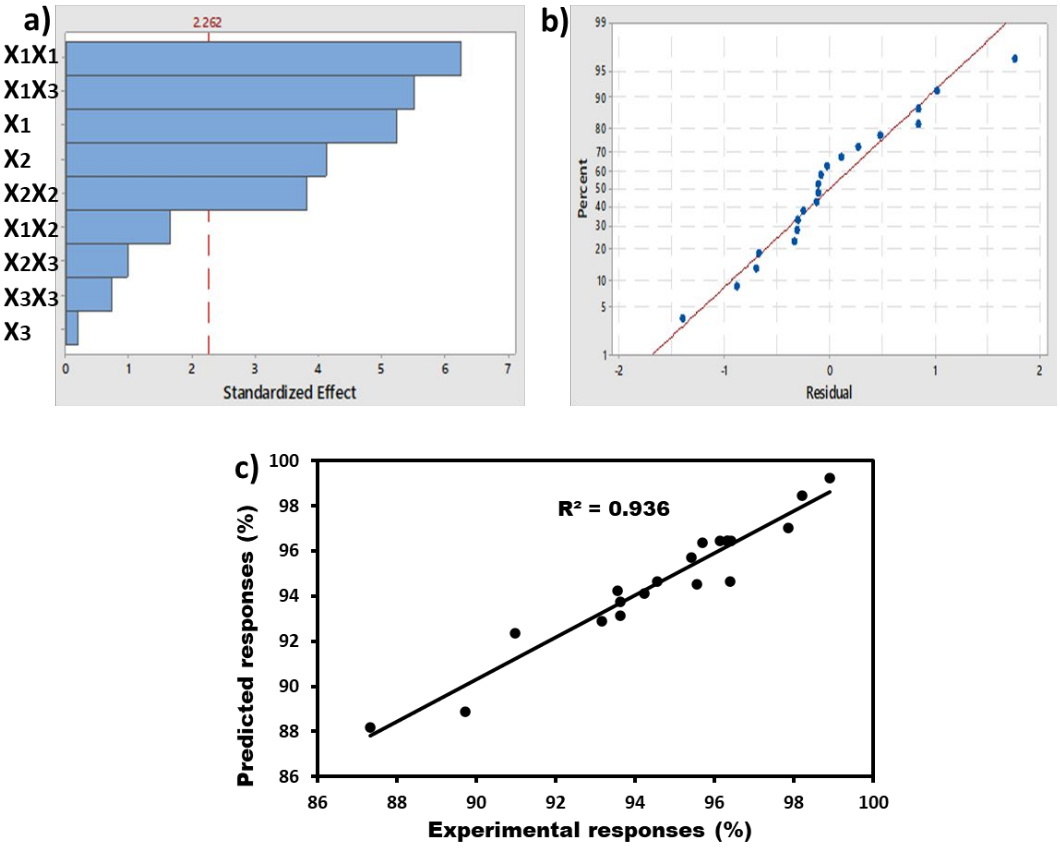

Figure S1. a) Standardised Pareto plot, b) normal probability curve for residues, and c) predicted responses versus experimental responses curve.

Table S2 indicates the absorption kinetic parameters used for the MO removal on the GBN11 adsorbent. The maximum experimental capacity of the adsorption process was 322.52 mg/g. Based on the correlation coefficients of the three kinetic models studied in this work, the pseudo-second-order model describes well the adsorption kinetics of the MO molecules onto the GBN11 sorbent. Table S3 shows the fitting results for selected isotherm models. The

Langmuir isotherm describes the adsorption process of the MO molecules on the GBN 11 surface. Thus, the adsorption of MO dye onto the surface of the sorbent is controlled by the monolayer process.

Table S2. Adsorption kinetic parameters for MO on GBN 11

Kinetic model		MO
	$q_{e\ (exp)}$ ($mg.g^{-1}$)	322.52
Pseudo-first order	$q_{e\ (exp)}$ ($mg.g^{-1}$)	489.215
	k_1 (min^{-1})	0.7597
	R^2	0.8982
Pseudo-second order	$q_{e\ (exp)}$ ($mg.g^{-1}$)	140.8450
	k_2 ($g.mg^{-1}.min^{-1}$)	0.0336
	R^2	0.999
Intraparticle diffusion	C ($mg.g^{-1}$)	130.68
	$k_{id,1}$ ($mg.g^{-1}.min^{-1/2}$)	1.3755
	R^2	0.743

Table S3. Fitting results for some isotherm models

Isotherms	Parameters	MO
Langmuir	q_e (mg/g)	58.139
	K_L ($L.g^{-1}$)	21.5
	R^2	0.9927
Freundlich	K_F (mg/g)	38.861
	n	1.622
	R^2	0.9140
Temkin	K_r (L/mg)	1.4753
	B	33.2886
	R^2	0.9716
